# Recurring alcohol-related care between 1998 and 2007 among people treated for an alcohol-related disorder in 1997: A register study in Stockholm County

**DOI:** 10.1186/1471-2458-11-574

**Published:** 2011-07-19

**Authors:** Kozma Ahacic, Kerstin Damström-Thakker, Ingemar Kåreholt

**Affiliations:** 1 Karolinska Institutet, Social medicine, Department of Public Health Sciences, Karolinska Institutet, Box 170 70, SE - 104 62 Stockholm, Sweden; 2 Karolinska Institutet, Applied Public Health, Department of Public Health Sciences, Karolinska Institutet, Box 170 70, SE - 104 62 Stockholm, Sweden; 3 Aging Research Center, Karolinska Institutet & Stockholm University, Gävlegatan 16, SE - 113 39 Stockholm, Sweden

**Keywords:** recidivism, relapse, treatment, revolving door, rehospitalization

## Abstract

**Background:**

Inpatient care for alcohol intoxication is increasing in Sweden, especially among young women. Since it is well known that alcohol disorder is a chronic relapsing illness, this study examines the extent to which people return for more care.

**Method:**

All inpatients with alcohol-related diagnoses in Stockholm County during 1997 were followed prospectively to 2007 through registers. The proportion reappearing for the same diagnosis, other alcohol-related inpatient, or outpatient care each year after baseline, as well as the number of years the inpatients reappeared were calculated (n = 2735). Three diagnoses were examined separately; alcohol dependence, harmful use of alcohol, and alcohol intoxication.

**Results:**

Three out of five inpatients with an alcohol diagnoses reappeared for more alcohol-related inpatient care during the following decade. The proportion returning was largest the year after baseline and then decreased curvilinearly over time. The inclusion of outpatient care increased proportions, but did not change patterns. Of those with an alcohol dependence diagnosis at baseline 42 percent returned for more alcohol-related inpatient care the first, 28 percent the fifth, and 25 percent the tenth year. Corresponding proportions for harmful use and intoxication were smaller. One in five among those with an alcohol dependence returned for more than five of the ten years. Ordered logistic regressions confirmed that besides diagnosis, age and gender were independently related to the number of years returning to care.

**Conclusions:**

While middle-aged males with alcohol dependence were in a revolving door, young female inpatients with intoxication diagnosis returned to a comparably lower degree.

## Background

There is a fear that an inpatient episode with an alcohol diagnosis, especially among younger persons, represents a serious step towards a career of repeated alcohol-related hospitalizations. It is well known that alcohol disorder is a chronic relapsing illness, e.g. see [1,2]. Moreover, in Stockholm between 1997 and 2007 the number of persons who received inpatient care for alcohol intoxication during a year in ages 15-24 years more than doubled among men while it showed a threefold increase among women [3]. Although chronic relapsing illness has been a phenomenon well studied within mental health care [4-11], few studies seem to have examined the extent to which people reappear for more alcohol-related health care [12-17].

Such analyses may have been done historically, locally, and may not have been published internationally; this was largely the case for Sweden [12-16]. Already in 1964 a Swedish government report showed that 82 percent of all male clients at the Temperance Board had received care previously [12]. Fifty percent had received care within the previous two years, and 72 percent within four years. In 1974 a Swedish government report showed that 14 percent of the persons with an alcohol dependence diagnosis who were admitted into mental health care had had four or more earlier care episodes [13]. Similarly, in 1978, 27 percent of all 'inebriates' (n = 7,686) admitted to public and private institutions had been previously admitted four times or more [14]. A recent study in Stockholm County showed that the odds of being in treatment among those who had been in treatment the previous year were 18 times greater - even after adjusting for various background conditions [16]. A study of US war veterans similarly indicated that earlier care predicted later care [17]. However, even if these previous studies have reported on the importance of treatment history, this has not been easily translated into a prospective outlook or risk assessment.

In order to study the extent to which people are being rehospitalized we decided to follow a cohort of Stockholm County's patients through the registers. In Sweden all health care is registered. The present ICD-10 system of diagnoses was introduced here in 1997 [18], and the year therefore presented an obvious baseline.

It is unclear whether the various alcohol-related diagnoses predict recurrent hospitalizations differently. In Stockholm County alcohol intoxication, harmful use of alcohol, and alcohol dependence have been the three most prevalent alcohol-related diagnoses [3]. While there is a progressive severity indicated by these diagnoses, it takes time to develop dependence and diagnosing the disorder is not always straightforward. People can receive a diagnosis of alcohol intoxication at a young age, for example, due to one incident of excessive drinking. Physicians are probably more likely to diagnose harmful use for a less severe dependence, whereas the alcohol dependence diagnosis obviously includes the most severe cases. It therefore seems plausible that the patterns of readmission for these diagnoses differ, e.g. alcohol dependence as a diagnosis probably means higher odds for years of recurrent hospitalizations than an intoxication diagnosis. Whether this relationship is independent of age and gender is unclear. Women have previously been found to be less likely to be rehospitalized than men [19], and it seems likely that younger patients, being at the beginning of their alcohol career, are less likely to be rehospitalized than older patients.

The article aims to study the extent of recidivism in alcohol-related hospital care by following a cohort of patients over time. It examines the extent to which all inpatients with an alcohol-related diagnosis in Stockholm County during one year reappeared in inpatient or outpatient care over a ten-year period - and whether this varied according to diagnoses, age, and gender.

## Methods

### The Stockholm County registry

Stockholm County had 1.4 million inhabitants during the baseline year 1997. It is an expanding metropolitan/urban region. During 1997 the County registered approximately 5,400 patients with an alcohol-related inpatient care episode, and the study's follow-up took place during a time period of outpatient care expansion. In addition to the health care system, there is the social service system, including general support, specialized treatment programmes, and institutional care for longer periods.

The public health care sector includes detoxification and specialized treatment programmes. The County provides two specialist emergency units for addictive diseases to care for patients with acute substance-related conditions, i.e. mainly detoxification. While some patients may be the responsibility of other hospital and emergency departments, most patients with an urgent alcohol-related need of medical or psychiatric attention are served by these specialist units.

If intoxicated persons seeking urgent care at a hospital are assessed by the staff not to need primarily somatic care, they may be transferred to one of these two units. People suffering from alcohol intoxication also seek care at these two units directly, and others are brought in by police, for "sobering-up" or detoxification, around the clock. When patients need very strict supervision, or more than 6 hours to become sober, they are transferred to an inpatient area. Patients with a high risk of neuropsychological conditions, such as withdrawal seizures and alcohol withdrawal delirium, may also be transferred to a designated inpatient ward for prolonged care and observation.

Admission treatment data have been obtained from the Stockholm County Inpatient Care Register for the time period 1997 to 2007. These register data are considered reliable and have been previously used, among other things, to track time trends for alcoholic disorders, e.g. see [20-22].

### Sample inclusion criteria

The diagnoses were used both as inclusion criteria and for variable construction. They follow the International Classification of Diseases (ICD) 10th revision [18]. The study examines all alcohol-related diagnoses, but the three most prevalent conditions were also examined separately: alcohol intoxication, corresponding to F10.0, acute intoxication due to alcohol, or T51 toxic effect of alcohol; the harmful use of alcohol F10.1; and alcohol dependence F10.2. Having received one of the diagnoses does not preclude other diagnoses and treatments, and the journals therefore often include several diagnoses for each care episode. For inclusion in the study, the main diagnosis was considered alongside two contributory diagnoses. In the registers, the main diagnosis reflects what individuals usually received care for. Besides the above mentioned, the following alcohol induced or related diagnoses are included: alcohol-induced chronic pancreatitis K86.0, alcoholic liver disease K70, alcohol-induced pseudo-Cushing's syndrome E24.4, degeneration of nervous system due to alcohol G31.2, alcoholic polyneuropathy G62.1, alcoholic myopathy G72.1, alcoholic cardiomyopathy I42.6, alcoholic gastritis K29.2, maternal care for (suspected) damage to foetus from alcohol O35.4, foetus and newborn affected by maternal use of alcohol P04.3, foetal alcohol syndrome Q86.0, blood alcohol level Y90, alcohol intoxication Y91, alcohol rehabilitation Z50.2, alcohol abuse counselling and surveillance Z71.4, mental and behavioural disorders due to use of alcohol (F10); withdrawal state F10.3; delirium F10.4; psychotic disorder F10.5 & F10.7, amnesic syndrome F10.6, other mental and behavioural disorders F10.8 & F10.9. All the included diagnoses have been listed as alcohol-related by the Swedish National Board of Health and Welfare [22].

### The study sample

In Stockholm County during the baseline year 1997 5,371 persons received inpatient care for an alcohol-related diagnosis [3]. This corresponds to 0.37 percent of the population. There were 3,342 persons with alcohol dependence as the first, second or third diagnoses in inpatient care, 914 persons with harmful use of alcohol, and 885 with alcohol intoxication [3].

In this study's analysis, all who moved from the county or who died during the follow-up period were excluded, which left n = 2,735. The study population was somewhat younger, but corresponded reasonably with the total population in terms of diagnoses, sex, and age-distribution. (Figures from the excluded group are presented in parentheses.) Among all inpatients at baseline in 1997, the proportion with alcohol dependence was 60.4 (64.2) percent, harmful use of alcohol 17.7 (16.3) percent, and alcohol intoxication 21.7 (11.1) percent. None of the diagnostic categories were exclusive. People could have up to three diagnoses per care episode, and were usually admitted several times (1 to 17 admissions, with an average of 1.7 admissions) during the baseline year 1997. The most common combination of diagnoses was alcohol dependence and harmful use of alcohol 4.9 (6.0) percent, followed by alcohol dependence and alcohol intoxication 2.1 (1.8) percent. A half percent had the combination of harmful use of alcohol and alcohol intoxication. Of the included persons, 8.7 (18.0) percent had none of these diagnoses, alcohol dependence, harmful use of alcohol, or alcohol intoxication, while 0.5 (0.7) percent had received all three. The average age for all inpatients at baseline in 1997 was 43.5 (52.9) years, and 69.1 (75.9) percent were men. For those with dependence diagnoses, the average age was 47.5 (53.2) years and 71.2 (78.8) percent were men, for harmful use, these figures were 42.9 (50.8) years and 69.0 (74.4) percent men, and for alcohol intoxication 31.4 (39.3) years and 63.7 (67.5) percent men.

### Analysis

The study analysed the proportion of inpatients from 1997 that returned each year after baseline in the decade between 1998 and 2007, and the number of years with treatment episodes. A year was chosen as the unit to measure time, since the proportion of the population with alcohol-related care during a calendar year has been used consistently as a measure in epidemiological surveys. The number of years with returns may be seen as a crude measure of the time with "unsuccessful" care or treatments, i.e. one or several care episodes. The reverse, to stay away from health care after treatment may - for this study's population - be seen as a proxy for cure, the goal of successful health care. It is important to note that the measures do not identify the reason why the persons stayed away, i.e. whether it was because of decreased drinking problems or some other reason. Ideally alcohol habits should have been included, but since it was a register study this was not feasible. The analyses made no distinction between the up to three diagnoses included per care episode, which corresponds with earlier operationalization in epidemiological surveys in Sweden. Most importantly it makes the number of cases at the study's baseline comparable to that of earlier reports.

To analyse the aggregated pattern, we first examined the extent to which patients returned for the same diagnosis. A return for the same diagnosis meant that that the diagnosis, e.g. intoxication diagnosis, was present as the only, or as one of several, diagnoses in the registers at baseline - in at least one episode - and this was examined similarly for each of the ten years in the follow-up. Other alcohol-related episodes without the particular diagnosis were thus disregarded. A second analysis was then conducted examining the extent to which patients returned for any alcohol related diagnosis. Thirdly, for the sake of comparison, the proportion of yearly returns for further inpatient care was complemented with the returns to either inpatient or outpatient care at the two units for addictive diseases.

To analyse patterns within individuals, the number of years with recurrent care episodes for alcohol-related diagnoses, for the period 1998-2007 among those that got alcohol related inpatient care in 1997 were examined and then modeled for the above outcomes. Independent variables in the models were sex, age group, and the diagnoses: alcohol intoxication, harmful use of alcohol, and alcohol dependence. In a last step, interaction terms between the three diagnoses were also added. Since the outcomes had skewed distributions ordered logistic regressions were suitable to model the number of years with repeated care episodes. Chi-square score tests supported our proportional odds assumptions, that is, that the odds ratios (ORs) of the independent variables were the same over the different number of years of return.

We also examined some possible confounding due to comorbidity. Of the n = 2735 with alcohol related diagnoses, n = 294 also received substance use disorder diagnosis during the baseline year. Substance use disorder was included as controls in the last models, without affecting the other estimates. These results were therefore not presented.

## Results

**Figure 1:**
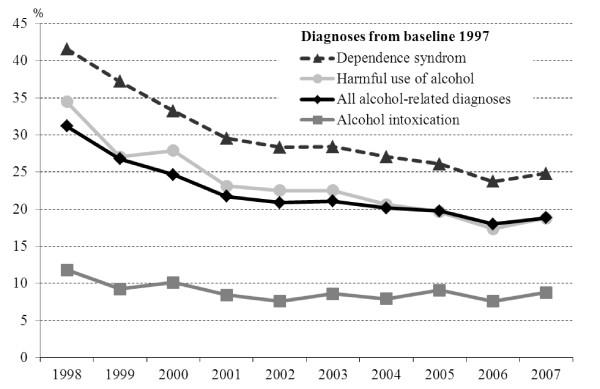
**Percentage of inpatients from 1997 who returned for inpatient care each year during the following decade; among those patients who had remained alive and continued to live in Stockholm County**.

Figure 1 shows the percentage of inpatients who returned for inpatient care each year after baseline. For alcohol dependence, approximately 40 percent returned for inpatient care in 1998, the first year after baseline. The graph is curvilinear, with a steeper initial decline followed by a gradual flattening out. In 2002 almost 30 percent returned, and in 2007 about 25 percent. The graph for harmful use indicates that approximately 35 percent returned for care the first year, around 23 percent in 2002, and almost 20 percent in 2007. The graph for alcohol intoxication indicates another pattern. Fewer returned in 1998, 12 percent, after that the graph continues on a rather straight course over time indicating a proportion slightly less than ten percent for each year.

**Figure 2:**
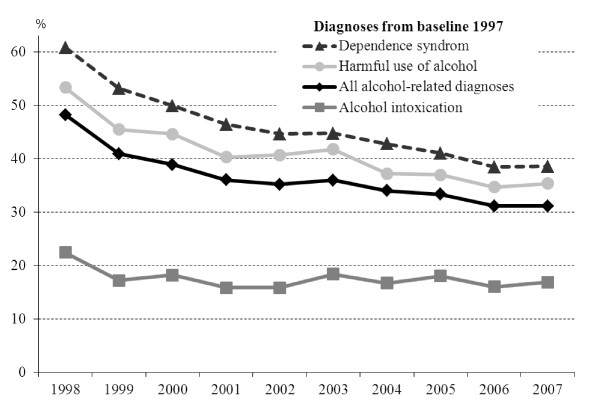
**Percentage of inpatients from 1997 who returned for in- or outpatient care each year during the following decade; among those patients who had remained alive and had continued to live in Stockholm County**.

In Figure 2 returns to outpatient care have been included, and the graphs thus show the proportion of inpatients that returned for inpatient and/or outpatient care. The graphs indicate similar patterns to those in Figure 1, but with proportions approximately one and half times larger.

### Number of years with recurrent care for alcohol-related diagnoses

Table 1 lists the number of years the inpatients returned during the decade after baseline. The first three rows show the risk of returning for the same diagnosis. A majority of the patients did not return for alcohol intoxication or harmful use, while half the patients with alcohol dependence returned two or more years with the same diagnosis. The next four rows show the risk of returning for inpatient care for the same or another alcohol-related diagnosis. Except for intoxication, the majority of inpatients returned at least one of the years. The likelihood of return was higher for the inpatients dependent on alcohol than it was for the alcohol-related inpatients in general. Those with a harmful use as a diagnosis were less likely and those who had received care for intoxication were least likely to return several times for treatment. About twenty percent with the dependent diagnosis returned for more than five out of the ten years. Out of all the inpatients with an alcohol-related disorder, 44 percent returned at least two out of the ten years.

**Table 1:** The number of years inpatients from 1997 returned for care over the following decade.

		1997 only -no return	Returned: 1 year	2-5 years	6-10 years	Total	
Diagnoses^1^		%	%	%	%	%	n^2^
**Returned with the same diagnosis**							
Alcohol intoxication		82	10	7	1	100	*593*
Harmful use of alcohol		61	19	19	1	100	*484*
Alcohol dependence		32	18	32	17	100	*1651*
**Returned for the same or another alcohol-related diagnosis**							
Alcohol intoxication		73	11	22	12	100	*593*
Harmful use of alcohol		38	17	30	15	100	*484*
Alcohol dependence		26	16	36	21	100	*1651*
Any alcohol-related diagnosis		41	15	29	15	100	*2735*
**Returned for inpatient or outpatient care**							
Alcohol intoxication		56	11	22	12	100	*593*
Harmful use of alcohol		20	12	33	35	100	*484*
Alcohol dependence		14	11	35	40	100	*1651*
Any alcohol-related diagnosis		27	11	32	30	100	*2735*
**Inpatients that returned for inpatient care**							
**Gender**	Men	39	14	29	17	100	*1891*
	Women	46	16	29	9	100	*844*
**Age group**	-24 years	88	6	5	1	100	*334*
	25-39	43	15	28	14	100	*578*
	40-49	29	17	34	20	100	*865*
	50-64	32	17	34	17	100	*818*
	65+	51	9	32	8	100	*136*
**Inpatients that returned for inpatient or outpatient care**							
**Gender**	Men	26	11	31	32	100	*1891*
	Women	29	13	33	26	100	*844*
**Age group**	-24 years	64	13	16	8	100	*334*
	25-39	25	10	30	36	100	*578*
	40-49	15	12	34	39	100	*865*
	50-64	21	12	38	29	100	*818*
	65+	44	11	29	21	100	*136*

^1 ^The categories of diagnoses are not exclusive.

^2 ^Adjusted for mortality and migration, i.e. those who died or moved away during the decade were subtracted from the total numbers.

Table 1 also indicates that the extent of returns increases when outpatient care is included. Furthermore, the Table shows that the percentage that returned differ by sex and age group. Forty-six percent of the men returned for care two or more years and for women the corresponding estimate was 38 percent. In the youngest age group up to 24 years, 6 percent returned two or more years, while in the age group 40-49 years, more than half of the patients returned two or more years for more alcohol-related inpatient care.

A further analysis, not presented, also shows that three percent of the (n = 123) female and seven percent of the (n = 211) male inpatients in the youngest age group, up to 24 years, returned two or more out of the ten years for further inpatient care. Twenty percent of the women and 25 percent of the men returned similarly for either in- or outpatient care. Out of the n = 264 (99 young women and 165 young men) with intoxication diagnoses in the youngest age group, 30 percent of the women and 28 percent of the men returned at least one year for either in- or outpatient care, and 16 percent among both young men and women did so two or more years.

Table 2 shows results from ordered logistic regressions presented as ORs for return to care for a higher number of years after baseline. Men had higher odds than women regarding returning for care; with the same diagnoses if the diagnosis was alcohol dependence but not otherwise, independently of age group. Men also had higher odds of returning for both inpatient care and in/or outpatient care independently of age group and diagnoses. The youngest age group, 13-24 years, had lower odds than the older age groups independently of gender for the same diagnoses and independently of gender and diagnosis for both inpatient and in/outpatient care (for in/outpatient care the odds for oldest age group 65+ was not significantly different from for the youngest). While people with intoxication diagnosis had lower odds, people with a harmful use - and similarly those with alcohol dependence - had higher odds of returning than those with other alcohol-related diagnoses for both inpatient and in/outpatient care.

**Table 2:** Odds ratios to return for more care during the following decade among all inpatients in 1997 treated for alcohol-related diagnoses, modeling the number of years the patients returned in ordered logistic regression models.

	For the same diagnoses			For		For	
	intoxication	harmful use	dependence	inpatient care		in- or outpatient care	
	OR 95% CI	OR 95% CI	OR 95% CI	OR^1^ 95% CI	OR 95% CI	OR 95% CI	OR 95% CI
**Sex**							
Females	1.0	1.0	1.0	1.0	1.0	1.0	1.0
Males	0.80	1.16	**1.60**	**1.39**	**1.38**	**1.18**	**1.17**
	*0.51-1.25*	*0.78-1.70*	*1.32-1.94*	*1.19-1.62*	*1.18-1.61*	*1.02-1.37*	*1.01-1.36*
**Age group**							
-24	1.0	1.0	1.0	1.0	1.0	1.0	1.0
25-39	**5.72**	**3.45**	**5.80**	**5.03**	**4.17**	**2.87**	**2.44**
	*3.06-10.7*	*1.42-8.40*	*1.88-17.9*	*3.43-7.39*	*2.82-6.18*	*2.16-3.81*	*1.82-3.27*
40-49	**6.14**	**4.78**	**8.70**	**7.06**	**5.64**	**3.00**	**2.50**
	*3.18-11.9*	*2.00-11.4*	*2.84-26.7*	*4.83-10.3*	*3.82-8.34*	*2.26-3.99*	*1.86-3.35*
50-64	**6.16**	**4.61**	**6.92**	**5.80**	**4.54**	**1.97**	**1.61**
	*2.97-12.8*	*1.90-11.2*	*2.26-21.2*	*3.94-8.52*	*3.06-6.74*	*1.47-2.63*	*1.19-2.17*
65+	2.16	**4.27**	**3.99**	**3.49**	**2.62**	0.85	0.68
	*0.44-10.6*	*1.21-15.0*	*1.23-13.0*	*2.16-5.64*	*1.61-4.28*	*0.57-1.28*	*0.45-1.03*
**Diagnoses**							
No intoxication diagnosis^1^				1.0	1.0	1.0	1.0
Alcohol intoxication				**0.73**	**0.32**	**0.64**	**0.37**
				*0.57-0.94*	*0.22-0.45*	*0.51-0.80*	*0.27-0.51*
No harmful use diagnosis^1^				1.0	1.0	1.0	1.0
Harmful use of alcohol				**1.74**	0.84	**1.98**	**1.40**
				*1.41-2.13*	*0.62-1.16*	*1.62-2.41*	*1.03-1.89*
No dependence diagnosis^1^				1.0	1.0	1.0	1.0
Alcohol dependence				**3.40**	**1.97**	**3.50**	**2.60**
				*2.80-4.12*	*1.53-2.54*	*2.90-4.23*	*2.04-3.33*
**2^nd ^order interactions - two diagnoses**							
Intoxication & harmful use				-	**9.58**(2.58)^2^	-	**6.37**(3.30)
					*3.48-26.3*		*2.37-17.1*
Intoxication & dependence				-	**4.55**(2.86)	-	**3.37**(3.34)
					*2.55-8.10*		*1.93-5.89*
Harmful use & dependence				-	**2.62**(4.34)	-	1.32 (4.80)
					*1.68-4.07*		*0.86-2.02*
**3^rd ^order interactions - three diagnoses**							
Intoxication, harmful use, & dependence				-	**0.14**(0.07)	-	**0.19**(0.26)
					*0.03-0.62*		*0.04-0.82*
*n*^3^	*593*	*484*	*1651*	*2735*	*2735*	*2735*	*2735*

^1 ^The OR 1.0 for the reference categories (i.e., the relative odds for the intercept), equals the average odds for all other diagnoses, i.e., all except those included in the model; intoxication, harmful use, and dependence.

^2 ^The ORs within parentheses show ORs in comparison to the reference category 1.0 (for other diagnoses) for the categories of people that had received two and three diagnoses, to complement the ORs for the interaction terms since these are not interpretable without recalculation.

^3 ^Adjusted for mortality and migration, i.e., those who died or moved away during the decade were subtracted from the total numbers.

The models with interaction terms suggest that having several diagnoses changed odds, that is, having two of the three diagnoses increased the odds in comparison to having one of them (the interaction between harmful use and dependence was not significant for in/outpatient care), while having all three studied diagnoses during the baseline year decreased the odds.

## Discussion

The readmission rate for alcohol-related care decreased over time: varying by diagnosis, the percentage that returned was largest the year following baseline and then declined gradually over time in a curvilinear fashion. Three out of five of the inpatients from 1997 reappeared at least once during the following decade, while two out of five reappeared at least two out of the ten years. One out of ten reappeared during more than five of the years.

The odds of returning varied by diagnosis, and having received several alcohol-related diagnoses (at the same or at different care episodes during the baseline year) also seems to have increased odds for yearly returns. At the same time, odds were lower for the patients (n = 32) who had received all the three diagnoses. The reason for this is unclear.

The results confirmed that being female, in the youngest age group, and having an alcohol intoxication diagnosis were independent factors that lowered odds for reappearing care. This was in line with a previous study showing that women have a lower risk for rehospitalization [19]. Our results also showed that gender was a predictor of repeated hospitalizations independently of age or diagnosis. While the previous study also showed that the risk was lower among those who received a shorter period of outpatient care after detoxification, the factors examined here, i.e. gender, age, and diagnosis, are not modifiable. The implications may therefore be less obvious, but at the same time basic descriptive approaches have previously been lacking. What other, preferably amendable, factors among individuals may contribute to decrease risk for repeated care may be an interesting subject for further study. Unfortunately, County registers contain little further information, e.g. no socioeconomic variables besides sex and age, which is a limitation. However, connecting registers to population surveys provides a promising future perspective.

The diagnoses in the registers are provided by the physicians, and our design included no specific validation of this system. To our knowledge there has not been any prior study about the validity of the system of diagnoses concerning alcohol-related diagnoses. Our analysis confirmed that diagnoses predict recurrent hospitalizations and that the three diagnoses; alcohol intoxication, harmful use of alcohol, and alcohol dependence, do so differently. The study can therefore be seen as providing some validity to the diagnostic system concerning alcohol as applied in health care, e.g. the study confirms that it was valid to see dependence as a more severe condition.

Previous studies have reported the importance of treatment history [12-17], and we have been able to complement earlier findings with a prospective outlook. Results confirm that alcohol dependence diagnosis meant an evident risk of being in a cycle of recidivism. In the Danish mental health care services approximately a tenth of the first-time patients were estimated to eventually become revolving door patients [23]. Revolving door patients were significantly younger than other patients and more likely to suffer from certain diagnoses, i.e. schizophrenia or alcohol/substance abuse [24].

To the extent that a diagnosis of schizophrenia is considered to be chronic, with respect to the need for care, alcohol dependence seems to be of the same dignity. Lien (2002) found the highest readmission rate in mental health care among schizophrenic patients, where 80 percent had been readmitted within 10 years [25]. In our study, 74 percent (86 percent if outpatient care is included) of those dependent on alcohol had been readmitted within a similar time frame. But then our study was based on a mixed cohort including patients already in a cycle of recidivism, while Lien studied patients from the first episode onwards.

The relationship between having the disorder and receiving professional help from health care is likely to differ between alcohol dependence and schizophrenia, and the comparison with schizophrenia may falter therefore also. Most people who change their alcohol habits are able to do so without professional help [26,27]. Some researchers have concluded that alcohol treatment serves as a place for handling a population of marginalized people [16]. Our results substantiate that it is a small group of people, probably marginalized in other respects also, who received care on a repeated basis.

People in treatment are more likely to be middle-aged, less educated, retired, on sick-leave, or unemployed, and to have unstable living situations. They drink more frequently and have more often been recommended by employers, co-workers, probation officer/court/lawyer, social services, or health care personnel to seek care [16]. In the future, given the availability of appropriate study materials, it should be interesting to prospectively examine the risk of ending up in the revolving door as an outcome separate from that of having an alcohol disorder or receiving treatment.

The possibility to return to care may be a good thing, but doing so also indicates continuing difficulties. In other words, there two sides to the coin, and a limitation is that it remains unclear what staying away from health care meant in this study. About half of the patients are likely to have decreased or quit drinking one year after treatment, as indicated by another study [28]. However, the long-term prognosis remains unclear. It would be desirable to examine the extent to which health care is able to help people change their alcohol habits, both over a longer time period, as well as in comparison to natural changes in the population [27]. Nonetheless, even if it is unclear what staying away meant in terms of drinking patterns, returning for more care does suggest a continuation of highly harmful drinking habits.

The decline of returns over the years in the decade following baseline presented in the figures reflects the aggregated likelihood of staying away from health care. The figure suggests that people did succeed in staying away - even if it took some time for some people. After a decade, nearly a third of the inpatients were coming back yearly. If the line is extrapolated, about the same proportion is indicated to come back yearly in the future also (given that they do not move away or die). In comparison, the odds of receiving alcohol or drug related in- or outpatient care in the general population in 2007 was about one and half percent [3].

The design with a mixed cohort was another limitation. In the studied cohort some of patients already had a history of care at baseline. For others it was their first visit. Obviously, the youngest age groups are the most likely to be at the beginning of their alcohol career. The youngest age group did reappear to a considerably lesser extent than older age groups. Whether this depended on their age or the fact that they are more likely to be closer to their début in their alcohol career remains unclear. Unfortunately, age and the numbers of years spent in an alcoholic career and the number of years on the pathway through the care system remain confounded in the study design. In future studies it would be desirable to follow the patients from their first episode of alcohol-related care onwards to see the extent to which and at what stage they reappear. Presently, it is clear that this mix of people at different stages of their alcohol-related health care career affected the results. A mixed cohort, where a substantial proportion of persons are already in a cycle of recidivism, is likely to overestimate the extent of recidivism in comparison to a cohort which is followed from the first care episode onwards.

It is important to realize that factors other than those on the individual level, i.e. patient characteristics, are likely to explain the level of care. This is likely to be true for initial visits as well as for the extent of rehospitalizations. The reorganization of mental health care in Sweden and elsewhere has moved from large institutions, where patients could remain for life, to revolving door care patterns. This demonstrates how structural factors, such as the organization of care, may influence the level of care. It also indicates that it may be difficult to generalize from the present study's results. The organization of alcohol-related care is likely to vary between countries, as well as over time.

Not least in Sweden, the organization of alcohol-related care has changed during the past century. This is also apparent from previous Swedish studies of the various authorities responsible for care [12-14,16]. During the studied period, there was a pronounced effort to launch outpatient instead of inpatient care. Stockholm County Council, responsible for health care, and the Confederation of Local Authorities, consisting of 26 municipalities in Stockholm County which provide social services, decided to reduce inpatient care and increase outpatient care in the 1990s. New local outpatient clinics were opened, and social services have collaborated with health care's specialized addiction services.

This was also why outpatient care was included in some of the analyses. It was not, however, possible to distinguish alcohol from drug-related outpatient care in these results. This is a limitation in the study. When readmission included outpatient care - in addition to all inpatient care - the estimated percentage returning yearly was higher, but the curvature showing the decrease of the percent over time was similar. While the majority of outpatient care episodes are likely to have been alcohol-related, it remains unclear how much the percentage would have decreased if it had been possible to exclude the drug-related episodes.

On the other hand, social services' interventions were not included in estimates, since they are not part of health care records. Including people who returned to treatment provided by social services is likely to have increased the number of returns, but to what degree is unknown. Nevertheless, diagnoses are only provided by the health care services.

Attrition and selective mortality do pose a challenge in cohort studies. Including attrition, i.e. those who moved - and coding the years away as years with no care episodes - did not affect the estimates of the proportion returning. This, in turn, suggests that those who moved had a higher degree of recidivism than the selected sub-sample. This is because they may have received care elsewhere, e.g. in another county, during the time they were not living in Stockholm. In other words, by excluding migration the degree of recidivism was possibly underestimated.

On the other hand, if people who died were included in the estimates - and the years they were dead coded as years with no care episodes - obviously fewer people would reappear. The estimated proportion for those with an alcohol dependence diagnosis at baseline to reappear for inpatient care for more than five of the years was 11 instead of 15 percent, when those who died were included. To examine the possible bias because of the mortality an alternative graph was calculated, including all those alive in the total for each year. These estimates suggest that those who died had a higher degree of recidivism. Although the proportion of inpatients that returned for more inpatient care were about same towards the end of the decade, 36 percent rather than 31 percent of the inpatients returned the first year after baseline.

We also estimated the corresponding bias regarding the independent variables in a similar way, i.e. by including those who migrated or died as non-returns. Analysing the models for repeated years of care and including those who moved or died after baseline as no-returns did not change the general picture, but gave somewhat lower ORs - particularly for the oldest age groups.

From a patient's perspective being alive and in the same place is likely to be the implicit assumption - if someone considers her/his risk of ending up in care again. Nonetheless, from an epidemiological perspective, death or moving away represents one of several explanations to why patients do not show up for care again. While in our study attrition, i.e. moving away seems to be of less influence in this respect, selective mortality deserves a separate study. Not least among the older ages, selective mortality did provide an explanation as to why persons did not reappear for alcohol-related care.

This study was based on register data. Whether this represents a limitation is not clear, the opposite may be as likely. People with severe drinking problems are probably less likely to appear in convenient sampling or as responders in randomized population surveys. Treatment studies, on the other hand, are often selective. Treatment may target those with insurance, with jobs at workplaces providing health care, or people with sufficient incentive. This may, for example, lead to a targeting of less severe cases. On the other hand, few population samples are large enough or systematically stratified in such a way to include a representative spectrum of people with drinking disorders of different severity. However, in diverse clinical populations, more stratified samples arise naturally. At the very least, our use of population-based health care records is likely to reduce the possibility of different selection effects.

## Conclusions

Young people and young women in particular have shown large increases in alcohol-related care consumption for alcohol intoxication during the past decade in Stockholm County. This study suggests that being young, female, and receiving an intoxication diagnosis were factors that independently lowered the risk for years of repeated care. Three out five patients who received inpatient care for an alcohol-related diagnosis came back for further inpatient care one or several years in the decade following a year with treatment. The study thus substantiates that there was an evident risk of being in a revolving door, but it also suggests that this risk was lower for those categories that exhibited large increases in alcohol-related care consumption during the past decade in Stockholm [3]. More research is needed to examine more closely the effects of early treatment during the life course.

## Conflict of Interest

The authors declare that they have no competing interests.

## Authors' contributions

KA conceived the study, performed the statistical analysis, and wrote the draft of the manuscript. KDT and IK revised it critically for important intellectual content and all authors read and approved the final manuscript.

## Pre-publication history

The pre-publication history for this paper can be accessed here:

http://www.biomedcentral.com/1471-2458/11/574/prepub
